# Quality of care associated with number of cases seen and self-reports of clinical competence for Japanese physicians-in-training in internal medicine

**DOI:** 10.1186/1472-6920-6-33

**Published:** 2006-06-13

**Authors:** Yasuaki Hayashino, Shunich Fukuhara, Kunihiko Matsui, Yoshinori Noguchi, Taro Minami, Dan Bertenthal, John W Peabody, Yoshitomo Mutoh, Yoshihiko Hirao, Kazuhiko Kikawa, Yohei Fukumoto, Junichiro Hayano, Teruo Ino, Umihiko Sawada, Jin Seino, Norio Higuma, Hiroyasu Ishimaru

**Affiliations:** 1 Kyoto University Graduate School of Medicine, Kyoto, Japan; 2 Kumamoto University Hospital, Kumamoto, Japan; 3 Nagoya Second Red Cross Hospital, Aichi, Japan; 4 Beth Israel Medical Center, NY, USA; 5 University of California, San Francisco and Los Angeles, California, USA; 6 Toranomon Hospital, Tokyo, Japan; 7 Nara Medical University, Nara, Japan; 8 Yamaguchi University Hospital, Yamaguchi, Japan; 9 Nagoya City University Medical School, Aichi, Japan; 10 Nihon University School of Medicine, Tokyo, Japan; 11 National Hospital Organization Sendai Medical Center, Niigara, Japan; 12 Niigata Municipal Hospital, Niigara, Japan; 13 Tenri Hospital, Nara, Japan

## Abstract

**Background:**

The extent of clinical exposure needed to ensure quality care has not been well determined during internal medicine training. We aimed to determine the association between clinical exposure (number of cases seen), self- reports of clinical competence, and type of institution (predictor variables) and quality of care (outcome variable) as measured by clinical vignettes.

**Methods:**

Cross-sectional study using univariate and multivariate linear analyses in 11 teaching hospitals in Japan. Participants were physicians-in-training in internal medicine departments. Main outcome measure was standardized t-scores (quality of care) derived from responses to five clinical vignettes.

**Results:**

Of the 375 eligible participants, 263 (70.1%) completed the vignettes. Most were in their first (57.8%) and second year (28.5%) of training; on average, the participants were 1.8 years (range = 1–8) after graduation. Two thirds of the participants (68.8%) worked in university-affiliated teaching hospitals. The median number of cases seen was 210 (range = 10–11400). Greater exposure to cases (p = 0.0005), higher self-reports of clinical competence (p = 0.0095), and type of institution (p < 0.0001) were significantly associated with higher quality of care, using a multivariate linear model and adjusting for the remaining factors. Quality of care rapidly increased for the first 100 to 200 cases seen and tapered thereafter.

**Conclusion:**

The amount of clinical exposure and levels of self-reports of clinical competence, not years after graduation, were positively associated with quality of care, adjusting for the remaining factors. The learning curve tapered after about 200 cases.

## Background

Healthcare systems throughout the world are searching for better ways of delivering high quality care. Attention to quality of patient care has become an important healthcare issue during the last decade, not only for health authorities, policymakers, and managers, but also for physicians and patients. Improving the quality of healthcare involves a broad range of discrete activities such as rigorous evaluation of conventional treatments, incorporating patients' views in healthcare decisions, and audit and feedback of healthcare practices. Physicians are one of the main healthcare providers and are confronted with increasing pressure to provide and improve care. The skills and knowledge of physicians improve through a combination of didactic and experiential learning that can in turn contribute to improving patient care [1]. Learning occurs through repeated experience with many clinical cases. The number of clinical cases seen might be an important factor linked to quality of care.

The number of clinical cases needed to meet optimal levels of proficiency in surgical procedures [2,3], such as colonoscopy [4], has been evaluated often. However, there is very little literature that evaluates the impact of the amount of clinical exposure on quality of care in internal medicine, that is, the learning curve, especially for residents [5]. Bugelski suggested in the 1970's that the major increment in learning occurs in the early stages of exposure and that less is learned during later stages [6]. This theory has been tested especially for various surgical procedures and invasive diagnostic tests [2,4,7], but there was only one study (Medline search) that examined the learning curve for internal medicine training. Day et al. concluded that the rate of increase in self-reports of clinical competence in specific skills was influenced by the number of post-graduation years [5]. Residents in the first post-graduation year (PGY1) reported that their skills improved by an average of 196% during that year compared to less than 50% for residents in the third year. The goal of this study was to examine the relationship between self-estimates of clinical exposure (number of cases seen) and self-reports of clinical competence and quality of care using specially designed clinical vignettes.

## Methods

### Sample

An anonymous survey was administered in departments of internal medicine at 11 teaching hospitals in Japan, a convenience sample from 6 university-affiliated and 5 non-affiliated teaching hospitals (with oversampling of university settings). All physicians-in-training within 10 years of graduation from medical school were eligible for the study (n = 375). To avoid contamination, the survey was administered on the same day (03/14/2003) in all institutions.

### Measurement method

Quality of care was defined as the delivery of patient care in a manner that leads to better outcomes for individuals and populations [8]. Clinical vignettes have been used to measure variations in quality of care [9]. The scores derived from the vignettes reliably reflected actual levels of physician practice, resulting in higher criterion validity compared to scores derived from chart abstractions. Based on disease prevalence in Japan, we began by selecting six clinical vignettes to measure quality of care: four common outpatient chronic conditions (diabetes mellitus, chronic obstructive pulmonary disease, vascular disease, and depression) and two acute emergency room conditions (subarachnoid hemorrhage and gastrointestinal bleeding).

Two detailed clinical vignettes were developed for each chronic condition, for a total of 8 vignettes. These vignettes were originally developed to measure quality of care in the United States. The vignettes were translated in Japanese and partly revised to match clinical practice in Japan, for example, using equivalent drugs and screening procedures. In addition, we developed two original Japanese vignettes for the two acute conditions. From the 10 vignettes available, each participant received five randomly selected vignettes, one from each condition (4 chronic and 1 acute). The vignettes required open-ended responses to questions that were presented in sections characteristic of a typical patient encounter: presenting complaint, history, physical examination, radiological or laboratory tests, diagnosis, and treatment and management plans. Each section began with the presentation of new information. After answering a given section, participants could not return to previous sections to revise (possibly improve) their answers. Participants were given 85 minutes to complete all 5 vignettes.

Clinical exposure was measured using participant self-estimates of the number of patients seen in in-patient wards, outpatient clinics, and emergency rooms. Data was also collected on the number of years after graduation, type of institution (university-affiliated teaching hospitals or non-affiliated), self-reports of clinical competence (i.e., problem-solving ability, basic procedural skills [e.g., venipuncture, bone marrow aspiration], and basic medical knowledge), and communication ability (i.e., attitude toward patients and their family and cooperativeness with other medical staff). Self-reports were rated using a five-point ordinal rating scale (i.e., unsatisfactory, satisfactory, good, excellent, or outstanding). The overall model consisted of one quality-of-care outcome variable, portrayed by the vignettes, and four predictor variables, that is, self-estimates of total number of patients seen, type of institution, and self-reports of clinical competence and communication ability. The latter two variables were also summed to create a global self-reported competence variable.

### Scoring

The responses to the vignettes were scored by the authors. To ensure consistency in scoring, given conditions were scored by the same author. With regard to chronic conditions, we used the scoring criteria developed by the original American authors who based their criteria on national guidelines [9]. These criteria were then reviewed and ratified by expert panels of academic and community physicians in Japan, in fields relevant to each condition; in the end the original criteria were adopted. Scoring criteria for the acute conditions were developed de novo, using expert panels of Japanese physicians. To verify the equivalence of the Japanese version with the original English version, the 10 vignettes were back-translated into English and verified by the original American authors. Based on their recommendations and consensus among the authors, the vignettes and scoring criteria were finalized. Each vignette contained an average of 37 criteria (range = 26–50). Each criterion was rated according to a three-level quality-of-care scale: adequate, unnecessary, and inappropriate care. A one-point credit was assigned for each criterion when adequate care was proposed. An overall vignette score was assigned by summing the scores from the individual criteria.

First, we used the general linear model to test for a vignette (disease condition) effect; there was no such effect (p = 0.239). Thus the scores from all five vignettes were added for each participant and then converted to a standardized t-score with a mean of 50 and a standard deviation of 10; t-scores were used as the outcome (criterion) variable for quality of care in the analyses. This transformation facilitated the interpretation of the relative importance of the predictor variables by comparing the corresponding t-scores to means of 50.

### Predictor variables

The amount of clinical exposure was computed by adding the number of cases that each subject had seen in each setting (inpatient, outpatient and ER) and then scored according to five ordinal categories: 0–100, 201–300, 301–400, 401–600, 601–800, >800 cases. The data distribution was skewed to the left and consequently ordinal categories were used because they fit the model better than log transformations (based on Akaike's information criteria – AIC [10]). We also used a broader range (200 vs. 100) for numbers above 401 because the data were skewed and sparse in those categories. The proportion of cases in the in-patient setting was also calculated and incorporated into the analyses because training occurs mostly in in-patient settings in Japan. The number of years after graduation could influence the amount of clinical exposure and was thus incorporated into the analysis using three ordinal categories (because again they fit the model better than log transformations): one year after graduation (PGY1); two years after graduation (PGY2); and more than 3 years after graduation (≥PGY3).

In addition to examining the relationship between overall clinical exposure and t-scores, we also looked specifically at the exposure to disorders similar to the ones in the vignettes. This was measured using a common disease index (CDI), defined as (numerator:) the number of cases seen that were similar to the diseases in the vignettes (i.e., stroke, gastrointestinal bleeding, COPD, heart failure, ischemic heart disease, depression, and diabetes mellitus), divided by (denominator:) the total number of cases seen. The t-scores were plotted against CDI to interpret graphically the relationship between the CDI and t-scores. (a measure of quality of care). We also examined the relationship between CDI and quality of care, adjusting for overall clinical exposure in order to verify whether simply increasing the proportion of clinical exposure to similar disorders would lead to higher quality for a fixed amount of exposure.

Clinical competence was defined as the sum of the self-assessed ratings for the three elements of competence (i.e., problem-solving ability, basic procedural skills, and basic medical knowledge). CDI and clinical competence scores were each further divided into three-level variables: low (up to 33^rd ^percentile), middle (up to 67^th ^percentile), and high (greater than 67^th ^percentile). The maximum score within each level of competence was 15, that is, the sum of 5 points maximum for each element of competence (e.g., problem solving, procedural skills, and knowledge).

Quality of care can vary depending on the type of institution [9], and thus it was also incorporated into the analyses. Two types of teaching hospitals were included: university-affiliated and non-university-affiliated (community) teaching hospitals. While this variable was included in the analyses, specific nominal results about this factor are not reported because some hospitals did not consent to revealing type of institution.

### Analyses

Descriptive statistics included rates and proportions for categorical data and means and standard deviations (SD) for continuous data. We first performed univariate analyses to evaluate the relationship between predictor variables and quality of care. Analysis of covariance or pooled t-test was used for categorical data. Pearson or Spearman correlation coefficients were used for continuous data.

Multivariate linear regression models were then constructed to examine the association between clinical exposure and quality of care. We incorporated all predictor variables into the model because all of the variables were thought to be important factors that could potentially be associated with levels of quality of care. We tested the interaction between the amount of clinical exposure and type of institution and self-reports of clinical competence as well as for a case (vignette type) main effect. For all analyses, alpha was set at 0.05. Analyses were done using commercially available software (Intercooled STATA 8.0; STATA Corporation, TX, USA). Ethics approval was granted for this study by the Kyoto University Faculty of Medicine Institutional Review Board.

## Results

Of the 375 eligible physicians-in-training, 263 (70.1%) consented to participate, the majority of whom were first (57.8%) and second-year residents (28.5%). The mean number of years after graduation was 1.8 years (range: 1–8). Two thirds of the participants (68.8%) worked in university-affiliated hospitals. The median number of cases reported seen was 210 (range: 10–11400). The proportion of cases seen in inpatient settings was 48.6%, but the variance was large (SD = 27.4%). A third of the participants (34.7%) had seen more than 400 cases overall. See Table 1 for details.

**Figure 1:**
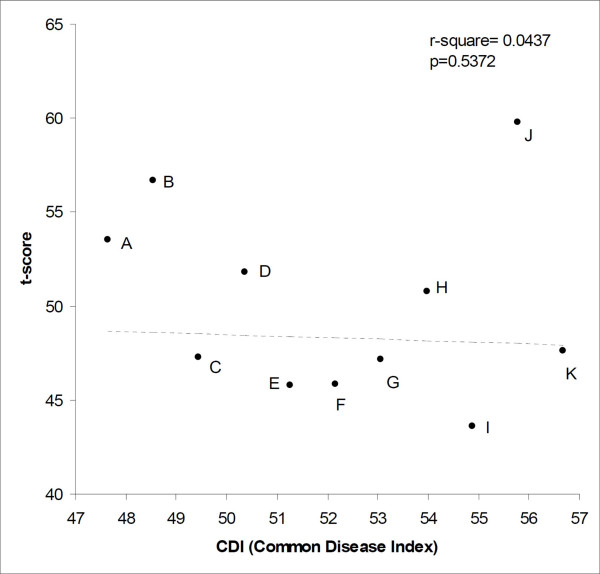
**Association between Common Disease Index (CDI) and t-scores**. Each point indicates the relationship between Common Disease Index (CDI) and the t-score for a given institution. The dotted line depicts the regression line (p = 0.5372).

Univariate analyses revealed that t-scores were significantly associated with the amount of clinical exposure, type of institution, number of years after graduation, and self-reports of clinical competence, but not with CDI, the proportion of inpatient cases, and self-reports of communication. The mean t-scores between types of institution were significantly different (p < .0001): 55.5 (SD = 2.1) compared to 47.5 (SD = 4.5) (the type of institution is concealed because certain institutions did not consent to revealing their identity). The mean t-scores increased with the number of post-graduation years, amount of exposure, and self-reports of clinical competence. Results of univariate and multivariate analyses are shown in Table 2. CDI was not associated with t-scores (Figure 1).

In the multivariate model, the number of post-graduation years was not statistically significant (p = 0.6942) while the proportion of inpatient cases seen was a statistically significant predictor (p = 0.0095). There was no significant interaction between the amount of clinical exposure and type of institution or levels of competence; consequently the interaction term was not included in the final model. CDI was not associated with t-scores, even after adjusting for the other factors.

**Figure 2:**
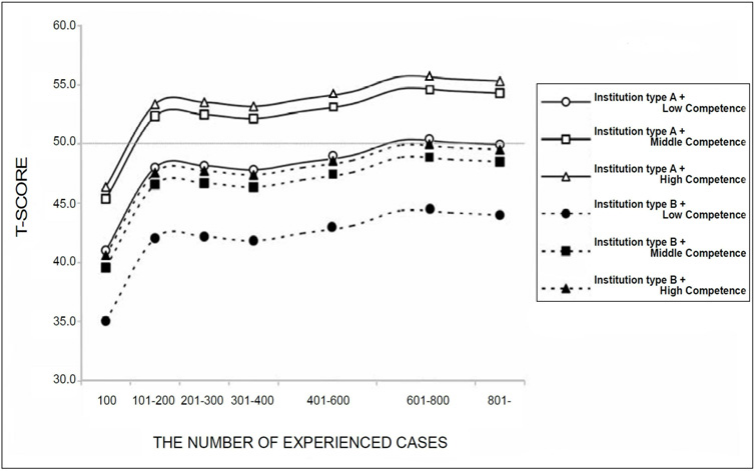
**Association between the Number of Cases Seen (clinical exposure) and Quality of Care, Stratified by Type of Institution and Levels of Self-reports of Clinical Competence, Adjusted for Other Factors**. Each curve indicates the relationship between the amount of clinical exposure and the t-score for each stratified group. Solid curves (A) indicate one type of institution (either university affiliated or non-university-affiliated teaching hospitals – identity kept confidential); dotted curves (B) indicate other type. Triangles indicate high competence levels; rectangles, middle competence levels; circles, low competence levels.

The slope of t-scores went up sharply for the first 100 to 200 cases and tapered thereafter. The t-scores were higher for one type of institution and for high competence levels, but the slope of the curves was the same among stratified groups, for given levels of amount of clinical exposure. The t-scores and amount of clinical exposure, stratified by type of institution and levels of self-reports of clinical competence, are graphically shown in Figure 2.

## Discussion

This study shows that quality of care for physicians-in-training in internal medicine in Japan increased as physicians saw more cases, especially during the initial stages, and tapered off thereafter. These results are consistent with those of Day et al. for PGY1 and PGY3 residents [5]. However, the number of years since graduation, which Day et al. suggested was an important predictor, was not significantly associated with quality of care when clinical exposure was included as a variable.

Although we have found that the overall amount of clinical exposure is an important determinant of the quality of care for physicians-in-training, a related problem is still unsolved: which of the amount of clinical exposure and quality of education is more strongly related to quality of care? To answer this question, we determine the association between the proportion having clinical exposure to certain diseases (CDI) and the quality of care (t-score) for those conditions, instead of evaluating the overall conditions. In the present study, the CDI was not associated with quality of care when adjusting for the overall amount of clinical exposure. Figure 1 illustrates this result. The CDI for institution B was among the lowest, indicating that physicians in that institution saw fewer similar diseases to the vignettes than physicians in other institutions. However, the average t-score for institution B was the second highest among all institutions. Although there is no accepted indicator for teaching, this institution is well known for its excellent teaching, and is the first educational hospital to have started residency training system in Japan; all authors agree that this hospital provides an excellent education. This suggests that physicians could have high quality of care in specific fields even if they had limited clinical exposure, if the quality of their education was excellent. The same point is made with Institution J, also known for its good teaching, which has a high t-score and high CDI.

There was a discrepancy between the effect on quality of care of the overall amount of exposure and the case-specific clinical exposure (CDI). The overall amount of clinical exposure was associated with better quality of care, whereas case-specific clinical exposure was not. A possible reason is that the most important skills to be acquired by internists in their training include basic skills and knowledge: history taking, physical examination, interpersonal skills, competence in continuing care, competence in diagnosis, selection of appropriate diagnostic studies, skills in searching evidence, clinical reasoning skills, and problem-solving skills [11,12]. These skills and knowledge could be acquired by experience in seeing various diseases, and could be applicable to any type of case seen subsequently, so that the amount of case-specific clinical experience is less important given the same amount of clinical exposure.

The present results indicate that self-reports of clinical competence are significantly associated with quality of care. However, some studies suggest that self-reports of competence are not in agreement with objective measures of clinical skills [13,14]. A possible explanation is misclassification of self-reports due to the anonymous (blinded) nature of the study. The participants might have considered that their self-assessments could influence their future career, so that they rated themselves higher than their actual performance. This hypothesis is supported by the observation of Woolliscroft et al. that the bottom quartile of medical students, according to objective evaluations, rate themselves higher than others [15]. Some other studies [14-16] did not state explicitly whether the self-reported ratings were blinded to evaluators. The anonymous self-reports were a strength of the present study, and this might have helped the participants make more accurate self-assessments with no resulting misclassification bias.

A limitation of the present study is that we did not adjust for medical school provenance or academic performance. Some studies suggest that selection of a medical school may influence practice outcomes [17,18]. This type of adjustment is important because of variations in the quality of education among schools and also because high achievers prefer to be educated in an institution with acknowledged excellent teaching. This could confuse the relation between type of institution and quality of care. Instead we adjusted for self-reported levels of competence, because we believe that the effect of current (self-assessed) competence was greater than that of past educational experiences.

## Conclusion

In summary, the overall amount of clinical exposure (number of cases seen) and levels of self-reports of clinical competence, but not the number of years after graduation, were significantly associated with quality of care, after adjusting for the remaining factors. Quality of education (e.g., the number and quality of the faculty) should be taken into account in future studies. It is possible that a selection bias exists whereby better quality students apply to more highly rated institutions.

## Competing interests

Fukuhara Shunichi was partly supported by a grant-in-aid from the scientific fund of the Ministry of Health, Labor and Welfare in Japan.

## Authors' contributions

YH developed the Japanese version of clinical vignettes and coordinated the study with SF, KM, YN and TM. YH, SF, KM, YH, TM and JWP conceived the study, and participated in the study design. YH and DB performed statistical analysis and drafted the initial manuscript for journal submission and participated in revisions. JWP developed the original version of clinical vignettes, and contributed to the evaluation of back-translated Japanese version. YM, YH, KK, YF, JH, TI, US, JS, NH, and HI coordinated data collection at each sites. All authors read and approved the final manuscript.

## Pre-publication history

The pre-publication history for this paper can be accessed here:



## Figures and Tables

**Table 1:** Baseline characteristics of participants (n = 263), self-reports of clinical competence, and common disease index (CDI)

	**n**	**Percentage**
**Institution affiliation**		
Type A*	181	68.8%
Type B	82	31.2%
		
**Post-graduation year**		
PGY1	152	57.8%
PGY2	75	28.5%
PGY3 and beyond	36	13.7%
		
**Number of cases seen**		
0–100	55	25.8%
101–200	36	16.9%
201–300	28	13.1%
301–400	20	9.4%
401–600	28	13.1%
601–800	16	7.5%
>800	30	14.1%
Median (range)	210 (10–11400)	
		
**Proportion of in-patient cases seen**	48.6% (± 27.4%)	
		
**Self-reports of clinical competence **(/15)		
Low	70	26.6%
Middle	84	31.9%
High	109	41.4%
Median (range)	6.4 (0–14)	
		
**Common Disease Index (CDI)**		
Low	62	23.6%
Middle	85	32.3%
High	116	44.1%
Median (range)	32 (0–100)	

* Type of institution (i.e., university-affiliated or non-university-affiliated teaching hospitals) is kept confidential; thus A and B were used without revealing specific identity.

**Table 2:** Relationship between predictor variables and quality of care (t-scores) according to univariate and multivariable analyses

**Predictor Variable**	**Univariate Analysis**			**Multivariate Analysis**		
	
	**Mean t-score**	**2SD**	P Value*	**Mean t-score**	**2SD**	**P Value**
**Institution affiliation**			<0.0001			<0.0001
Type A^+^	55.5	2.1		40.8	5.9	
Type B	47.5	4.5		47.8	4.2	
**Post-graduation year**			0.0317			0.6942
PGY1	48.6	1.6	---	40.8	5.9	---
PGY2	51.0	4.4	0.09	39.9	11.1	0.468
PGY3 and beyond	53.6	5.3	0.007	41.5	8.8	0.992
**Number of cases seen**			<0.0001			0.0005
0–100	45.4	2.4	---	40.8	5.9	---
101–200	49.4	6.3	0.041	48.0	10.4	0.001
201–300	52.4	6.6	0.001	47.6	11.2	0.002
301–400	51.9	7.1	0.006	48.8	10.9	0.011
401–600	54.7	6.6	<0.0001	50.1	12.3	0.001
601–800	55.3	7.5	<0.0001	49.8	12.8	0.004
>800	53.9	6.5	<0.0001	45.4	9.5	0.01
**Proportion of in-patient cases**†	-0.907†		0.1882			0.009
**Self-reports of clinical competence **(/15)			<0.0001			0.0095
Low	45.7	2.3	---	40.8	5.9	---
Middle	49.8	5.4	0.009	45.4	9.5	0.013
High	52.9	5.3	<0.0001	46.4	9.5	0.002
**Common Disease Index (CDI)**			0.2287			0.4962
Low	51.8	2.5	---	40.8	5.9	---
Middle	49.9	5.9	0.247	40.2	9.2	0.718
High	49.1	5.7	0.087	42.0	9.7	0.543

+ Type of institution (i.e., university-affiliated or non-university-affiliated teaching hospitals) is kept confidential; thus A and B were used without revealing specific identity.

* P-values for student t-test and ANOVA

† Pearson correlation coefficient
